# Evaluating the Cost-Effectiveness of Folic Acid Versus Methyltetrahydrofolate in Preventing Preeclampsia in Makassar, Indonesia

**DOI:** 10.7759/cureus.56671

**Published:** 2024-03-21

**Authors:** Andi Maulana Kamri, Rachmat Kosman, Bayu Putra

**Affiliations:** 1 Clinical Pharmacy, Universitas Muslim Indonesia, Makassar, IDN; 2 Pharmacology, Universitas Muslim Indonesia, Makassar, IDN

**Keywords:** folic acid dose, folic acid for pregnant women, acer, icer, hypertension

## Abstract

Background: Preeclampsia is a condition of elevated blood pressure with proteinuria that occurs during pregnancy and is a complication of elevated blood pressure. It usually occurs after the 20th week of pregnancy. This could be fatal for the mother after birth and the baby if it occurs before birth. The goal of this study is to investigate the risk, cost-effectiveness, and effective doses of folic acid (B9).

Method: This study is an observational study with a cohort design and random simple sampling data collection. Data was collected from the Cahaya Ibu Pharmacy Store in Makassar from 164 patients.

Result: Chi-square statistical analysis data showed a significant risk relationship between pregnant women and the development of hypertension compared with nonpregnant patients, with a p-value of 0.001. Her risk level for the event reaches twice, as evidenced by her odds ratio reaching 6.9 times. The results of cost analytics showed folic acid had an equal effect on women's reduced risk of preeclampsia as methyltetrahydrofolate.

Conclusions: Using folic acid early in pregnancy planning is a great opportunity to reduce the incidence of preeclampsia. The ICER value was obtained at $0.293, so an increase in the cost of that amount was needed to improve the effectiveness of therapy by patients using folic acid compared to patients with methyltetrahydrofolate therapy. Therefore, folic acid is more cost-effective compared to methyltetrahydrofolate.

## Introduction

Preeclampsia is a pregnancy disorder characterised by elevated blood pressure and proteinuria, particularly in the final trimester [1]. It is characterised by elevated blood pressure that often manifests at or above 20 weeks of gestation. If this is allowed to happen before delivery, it might be deadly for both the mother and the child. Food consumption during pregnancy might be influenced by a number of factors [2]. It is crucial for expectant mothers to adhere to the maintenance of their health during their pregnancy [3]. Currently, very few mothers are aware that taking folic acid during pregnancy is beneficial. We will examine the efficacy of folic acid supplementation for early pregnancy in this study. This study will pave the way for raising folic acid awareness and usage before or at the start of pregnancy planning. The purpose of this study is to ascertain the impact of providing folic acid to expectant mothers and to ascertain the optimal dosage of folic acid that they should take [4].

According to a number of earlier studies, pregnant women with preeclampsia had significantly lower vitamin B12 concentrations than pregnant women without the condition at the same age. An intriguing finding was that folic acid supplementation had no effect on raising blood haemoglobin levels, which are a factor in the prevalence of anaemia in pregnant women. Nobody has, however, raised the possibility that taking folic acid and raising blood pressure are related. Meanwhile, pregnant women also have a particular concern over the occurrence of anaemia [5-7].

An analytical method called economic assessment is used to compare two or more methods in terms of costs and results. A cost-effectiveness analysis (CEA) is one type of example used in pharmacoeconomic research. For pharmaco-social research comparing two or more health interventions with varying degrees of efficacy, the relatively straightforward CEA method is frequently utilised. When a drug's average cost-effectiveness ratio (ACER) value is lower than another drug's and its incremental cost-effectiveness ratio (ICER) value indicates a rise in cost per unit of the outcome, the data are analysed using CEAs, which are based on ACER and ICER. In this instance, cost-effectiveness involves both cost optimisation and the lowest cost [8,9]. Some vitamins that play an important role in the process of pregnancy include iron, mecobalamin, and folic acid [10]. Folic acid is a B vitamin that plays a role in cell metabolism and DNA synthesis [11].

Therapeutic policymakers can find options that are more affordable for each clinical outcome they achieve by doing research that makes use of this comparison. The least expensive option for a given treatment goal may not always imply the most cost-effective one. The cost-effectiveness of treating patients with methyltetrahydrofolate and generic folate (B9) vitamins will then be analysed based on this study. Because there isn't much research on folic acid, the researchers believe this study will provide useful extra information for doctors and other healthcare professionals. The effect of folic acid administration on blood pressure in the final trimester of pregnancy and the optimal dosage for pregnant women to take in order to prevent preeclampsia were among the issues that became the focus of this study. Clinicians can use the research's benefits as a guide to determine which forms of folic acid are safe for expectant mothers to take in order to preserve their health throughout the pregnancy.

## Materials and methods

Research design

This study uses a cohort design, observational methodology, and retrospective data gathering. Random simple sampling was used to choose the sample, meaning that all patients who took folic acid would be included in the group. From August 1, 2022, to December 30, 2022, the study was carried out at the Cahaya Ibu pharmacy in Makassar, the country's capital. This approach is employed because the researcher has to validate the patient's vitamin history, and data collection cannot be done to immediately follow up with the patient. This research has received approval from the Ethical Clearance Committee of Universitas Muslim Indonesia (approval number: UMI022210568).

Population

Patients with a gestational age of seven to nine months, comprehensive drug usage data, blood pressure data, and historical use of folic acid in the first semester were among the inclusion criteria. A total of 164 patients, including those who objected to being included in the group, met the exclusion criteria. Patients who did not have blood pressure test results were excluded from this research. We counted ACER and ICER to determine cost-effectiveness, and a statistical programme containing the Pearson correlation test and Chi-Square analysis test was utilised for data analysis. The association between blood pressure, folic acid dosage, and folic acid use duration is also visualised through a graphical presentation of the distribution of data on the normalcy of the connection.

Data collection

The researchers employed the method of interviewing all patients to confirm data regarding a history of folic acid usage. Additionally, the researchers utilised the patients' medical records to retrieve clinical assessments, particularly data concerning blood pressure. Data retrieval was conducted through a nurse's assistant to ensure the accuracy of blood pressure checks, serving as a double-check. Statistical analysis was employed to identify relationships between each variable.

## Results

This research focuses on looking at the use of folic acid and the factors that influence the incidence of preeclampsia. Folic acid (B9) and methyltetrahydrofolate are the same folate derivatives that can be used as the mother's vitamin. Chi-square statistical analysis data showed that there was a significant risk relationship between being pregnant and the incidence of an increased risk of preeclampsia compared to patients who were not using folic acid, with a p-value <0.001. The risk level of the event reaches two times, as seen in the odds ratio reaching 6.9 times. The data analytics are shown in Table 1.

**Table 1:** Chi-square analysis between the patient's consumption of folic acid and blood pressure

Variable		Systolic blood pressure during 32 weeks of pregnancy				p-value	Odds ratio
		<120 mmHg		>130 mmHg			
		n	%	n	%		
Pregnant patient	Consuming folic acid	133	47	31	18.9	<0.001	6.9
	Not consuming folic acid	2	6.5	29	93.5		

There are two stages to the placental illness of preeclampsia. An overabundance of anti-angiogenic factors is followed by an aberrant placentation in the first trimester, which is known as "motherhood syndrome" in late second and third pregnancies. The placenta's upregulation of hypoxia-inducible transcription factors and the presence of a gene profile linked with hypoxia point to hypoxia as a major aetiology of preeclampsia. Normal placentation results from hypoxia that follows oxygenation of the mother's bloodstream, and intermittent hypoxia and reoxygenation brought on by inadequate spiral arterial invasion might result in oxidative stress. Corin, a transmembrane enzyme that locally activates atrial natriuretic peptide by zymogen modification, may also have an impact on trophoblast invasion and remodelling of spiral arteries. The physiology of decidualization has a good understanding of uterine NK, which may have a role in the aberrant placentation seen in preeclampsia [12-15]. This was proven in the Pearson correlation test between the dose and the risk of blood pressure that might occur in patients. The results show that there is a significant relationship between reducing the risk of increasing blood pressure and the consumption of folic acid at doses >1000 µg, and the use of high doses of folic acid can reduce the risk of increasing blood pressure during the late trimester of pregnancy in women compared to patients who do not use folic acid or use folic acid. smaller doses of folate, such as 400 µg. The data analytics are shown in Table 2.

**Table 2:** Pearson correlation analysis between folic acid dose and blood pressure

Variable		Blood pressure				Pearson	p-value
		<120 mmHg		>130 mmHg			
		n	%	n	%		
Folate dose	1000 µg	100	98	2	2	r=0.505, n=133	<0.001
	400 µg	4	13.8	25	86.2		

The value of r=0.505 shows a positive correlation analysis with very strong correlation strength, besides the p-value, which shows a significant relationship between the use of high doses of folic acid and an increase in blood pressure. This is evidence that the use of folic acid at a dose of 1000 µg has clinical significance and a positive impact on reducing the risk of preeclampsia. It can be seen that the use of folic acid at a dose of 1000 µg and for more than two months during pregnancy planning is the golden time to reduce the incidence of increased blood pressure at the end of the trimester. Improvements in cost efficiency and additional costs are shown by the ICER value. In comparison to employing methyltetrahydrofolate, folic acid therapy requires an extra $0.293 in costs to improve the efficacy of preeclampsia prevention in one hypertensive patient against the treated group. When an intervention has a higher cost but a higher efficiency, ICER calculations ought to be performed. The data are shown in Table 3.

**Table 3:** ACER and ICER analytics for using folate acid ACER: average cost-effectiveness ratio, ICER: incremental cost-effectiveness ratio

Folic acid generation	Average cost of treatment	Average length of treatment	ACER		ICER
Methyltetrahydrofolate (4th generation)	$14.3	30 days	$1.22	$0.293	
Folic acid (B9)	$5.5	60 days	$0.09		

Consequently, the fourth generation of methyltetrahydrofolate was used in the computation of ACER values for the group of therapy users receiving folic acid. This comparison shows that the first generation of folic acid therapy groups is more cost-effective than the fourth generation of methyltetrahydrofolate folate therapy groups, which employ folate for at least two months prior to or during pregnancies, even at the same dose of 1000 mg [9]. Preeclampsia is characterised by the presence of proteinuria [1]. In another study, it was seen that there was a relationship between the incidence of mecobalamin deficiency during pregnancy and blood pressure disorders [6]. The vitamin needs of pregnant women during pregnancy will significantly differ each month. Folic acid is a supplement that can reduce the risk of preeclampsia. Folic acid can reduce placental perfusion in general and usually does not occur in the first and second trimesters. Folic acid can prevent and restore endothelial dysfunction; apart from plasma homocysteine, folic acid can also prevent increased blood pressure until the second stage. Therefore, folic acid can reduce the risk of preeclampsia occurring.

Preeclampsia continues to be a major global cause of maternal and newborn morbidity and mortality, and there is a continuous need to research efficient preventative measures. Early human and animal investigations have raised the possibility that folic acid may have a preventive effect against preeclampsia. We predicted that folic acid supplementation during pregnancy would reduce the risk of preeclampsia in a number of ways, including by promoting healthy placental growth and development in the first trimester, lowering blood homocysteine levels, and enhancing systemic endothelial function [5]. Consuming folic acid in the run-up to conception lowers the chance of neural tube abnormalities (NTDs). Among others, it mandated the folic acid fortification of white wheat flour and other enriched cereal products to lower the prevalence of NTDs. Since the introduction of fortification, the prevalence of NTDs has considerably decreased, and there is almost no evidence of widespread folate deficiency [7].

It is believed that serum folate can be transported to the placenta. Although it is advised, RBC folate screening for women is not yet common. According to Daly et al., RBC folate may be a stronger indicator of the risk of NTDs during the first trimester of pregnancy than serum folate. Within the first four weeks of supplementation, serum folate levels significantly increased in all of the women, although many of the participants (particularly in the 400 g/day group) did not reach an ideal RBC folate level. Only <20% of women failed to reach the desired RBC folate level after eight weeks when 800 g/day of folate was given. Women in both research groups with low baseline RBC folate concentrations (population median) continued to have lower concentrations than women with higher baseline values. Low doses need to be used for a longer period of time (4-8 weeks) than higher doses [16].

It takes folic acid as a cofactor to keep homocysteine levels within a reasonable range. Unbalances in serum vitamin B12 and folic acid (folic acid and vitamin B12) are more strongly linked to gestational diabetes mellitus. Folic acid is a crucial vitamin during pregnancy since it plays a role in DNA methylation and the manufacture of proteins and nucleic acids needed for cell replication and embryonic growth [17]. A safe and viable strategy that may provide pregnant women exposed to free radicals with a way to reduce the risk of foetal growth decline is higher-dose folic acid supplementation. The study's comparatively low attrition rate of 2% and strict adherence to the intention-to-treat principle are among its advantages [18].

## Discussion

Correlation between folic acid and preeclampsia

Folic acid is a fairly affordable supplement, making it an ideal choice for people from moderate to poor socioeconomic backgrounds. It has been demonstrated that folic acid consumption during pregnancy decreases aberrant trophoblast invasion into the uterus in patients with preeclampsia and does not impact neonatal weight as a result, resulting in a significant decrease in the number of postnatal cases requiring NICU care. Homocysteine levels, which are connected to the risk of preeclampsia, are also thought to be reduced by folic acid. Early to mid-pregnancy impaired placental perfusion marks the start of the first stage. Generalised endothelial dysfunction then follows, which is frequent in late pregnancy. Preeclampsia develops as a result of the final trimester. Reduced homocysteine release causes endothelial dysfunction, which folic acid can restore. This suggests that folic acid is crucial for pregnant women in their first and second trimesters. While folic acid has direct mechanisms to promote placental implantation and endothelial function, it also lowers homocysteine levels, which can lessen maternal endothelial damage and lower the incidence of preeclampsia [19-21].

Folic acid mechanism

The placenta and developing foetus have rapidly dividing cells, which increases the need for folate during pregnancy. Poor nutritional status and insufficient consumption of micronutrients before and throughout pregnancy have been associated with unfavourable pregnancy outcomes. Due to its role as a methyl donor in one-carbon metabolism and its significance in DNA synthesis, folate is essential for the growth of a healthy foetus and placenta during pregnancy. Low folate intake during pregnancy can inhibit one-carbon metabolism in vivo and reduce foetal and placental growth. Folic acid supplementation is believed to aid in the prevention of preeclampsia by ensuring an adequate supply of folate necessary for cell division, angiogenesis, trophoblast invasion, and endothelial-dependent arterial relaxation during pregnancy. Furthermore, homocysteine methylation, which lowers serum homocysteine levels during pregnancy, is facilitated by folate. Women with higher serum homocysteine concentrations have been found to have higher levels of the associated indicators, which are connected to endothelial dysfunction and oxidative stress in preeclampsia. Early in pregnancy, taking folic acid supplements offers a source of folate, which is essential for healthy placental implantation and blood supply. The reduction in maternal plasma folate levels later in pregnancy may be prevented by folic acid supplementation, ensuring that there is enough folate for the conversion of homocysteine to methionine. Folic acid dosage should be maintained after the first trimester because preeclampsia is known to be brought on by excessive plasma homocysteine levels [22-24]. The enzyme on which antifolates operate determines their mode of action. Even though there are many enzymes involved in the metabolism of folate, some of them stand out for their important contributions to folate's historical usage as a medication. Dihydropteroate synthase is one of the folate metabolism's most frequently studied enzymes. Despite the WHO's recommendation for a minimum dose of 400 µg, this dosage is exclusively for pregnant women to preserve their health [22,23,25].

## Conclusions

The use of high doses of folic acid, such as 1000 µg, and consumption before pregnancy or in the first trimester can reduce the risk of increased blood pressure events at the end of pregnancy. Even so, the use of folic acid early in pregnancy planning is a golden opportunity to reduce the incidence of preeclampsia. The results of the CEA with the lowest ACER were obtained for folic acid and methyltetrahydrofolate, so it can be concluded that folic acid is more cost-effective. Based on the ICER calculation results, it was obtained at $0.293, so an increase in the cost of that amount was needed to improve the effectiveness of therapy by patients using folic acid compared to patients with methyltetrahydrofolate therapy.
